# Analysis of Memory B Cell Responses and Isolation of Novel Monoclonal Antibodies with Neutralizing Breadth from HIV-1-Infected Individuals

**DOI:** 10.1371/journal.pone.0008805

**Published:** 2010-01-20

**Authors:** Davide Corti, Johannes P. M. Langedijk, Andreas Hinz, Michael S. Seaman, Fabrizia Vanzetta, Blanca M. Fernandez-Rodriguez, Chiara Silacci, Debora Pinna, David Jarrossay, Sunita Balla-Jhagjhoorsingh, Betty Willems, Maria J. Zekveld, Hanna Dreja, Eithne O'Sullivan, Corinna Pade, Chloe Orkin, Simon A. Jeffs, David C. Montefiori, David Davis, Winfried Weissenhorn, Áine McKnight, Jonathan L. Heeney, Federica Sallusto, Quentin J. Sattentau, Robin A. Weiss, Antonio Lanzavecchia

**Affiliations:** 1 Institute for Research in Biomedicine, Bellinzona, Switzerland; 2 Pepscan Therapeutics BV, Lelystad, Netherlands; 3 Unit for Virus Host Cell Interaction, UMI 3265 UJF-EMBL-CNRS, Grenoble, France; 4 Division of Viral Pathogenesis, Beth Israel Deaconess Medical Center, Boston, Massachusetts, United States of America; 5 Institute of Tropical Medicine, Antwerp, Belgium; 6 Queen Mary University, London, United Kingdom; 7 Barts and the London NHS Trust, London, United Kingdom; 8 Department of Infectious Diseases, The Wright-Fleming Institute, Imperial College Faculty of Medicine, London, United Kingdom; 9 Duke University Medical Center, Durham, North Carolina, United States of America; 10 Department of Virology, Biomedical Primate Research Centre, Rijswijk, Netherlands; 11 Department of Veterinary Medicine, University of Cambridge, Cambridge, United Kingdom; 12 The Sir William Dunn School of Pathology, University of Oxford, Oxford, United Kingdom; 13 Division of Infection and Immunity, University College London, London, United Kingdom; New York University, United States of America

## Abstract

**Background:**

The isolation of human monoclonal antibodies (mAbs) that neutralize a broad spectrum of primary HIV-1 isolates and the characterization of the human neutralizing antibody B cell response to HIV-1 infection are important goals that are central to the design of an effective antibody-based vaccine.

**Methods and Findings:**

We immortalized IgG^+^ memory B cells from individuals infected with diverse clades of HIV-1 and selected on the basis of plasma neutralization profiles that were cross-clade and relatively potent. Culture supernatants were screened using various recombinant forms of the envelope glycoproteins (Env) in multiple parallel assays. We isolated 58 mAbs that were mapped to different Env surfaces, most of which showed neutralizing activity. One mAb in particular (HJ16) specific for a novel epitope proximal to the CD4 binding site on gp120 selectively neutralized a multi-clade panel of Tier-2 HIV-1 pseudoviruses, and demonstrated reactivity that was comparable in breadth, but distinct in neutralization specificity, to that of the other CD4 binding site-specific neutralizing mAb b12. A second mAb (HGN194) bound a conserved epitope in the V3 crown and neutralized all Tier-1 and a proportion of Tier-2 pseudoviruses tested, irrespective of clade. A third mAb (HK20) with broad neutralizing activity, particularly as a Fab fragment, recognized a highly conserved epitope in the HR-1 region of gp41, but showed striking assay-dependent selectivity in its activity.

**Conclusions:**

This study reveals that by using appropriate screening methods, a large proportion of memory B cells can be isolated that produce mAbs with HIV-1 neutralizing activity. Three of these mAbs show unusual breadth of neutralization and therefore add to the current panel of HIV-1 neutralizing antibodies with potential for passive protection and template-based vaccine design.

## Introduction

Neutralizing antibodies provide one arm of the adaptive immune response against the human immunodeficiency virus type 1 (HIV-1). Several reports demonstrated that the neutralizing antibody response exerts selective pressure during HIV-1 replication *in vivo*, which accounts in part for the extensive variation in the *env* gene observed soon after primary infection [1], [2]. Furthermore, selective pressure imposed by neutralizing antibodies has been demonstrated in a human trial where three neutralizing monoclonal antibodies (mAbs) administered during HAART treatment-interruption led to a reduction in viremia followed by selection of escape mutants [3], [4]. Passive transfer studies in macaques showed that the administration of HIV-1 neutralizing mAbs protects against vaginal or intravenous challenge with SIV-HIV-1 chimeric viruses (SHIV) [5], [6], [7], [8], [9]. In some models protection depended not only on viral neutralization but also on Fc-mediated antibody effector functions [10], [11].

Given the predicted low-titer inoculum driving HIV-1 sexual transmission, a vaccine capable of eliciting antibodies that neutralize a broad spectrum of viral strains could potentially reduce or prevent infection. It has been anticipated that the identification of broadly neutralizing mAbs from HIV-1 infected individuals, and the characterization of their cognate epitopes will be instrumental in the design of immunogens capable of eliciting such a broad neutralizing response [12]. This idea has led to a major international cooperative effort within consortia of laboratories with complementary expertise in human immunology, structural biology and vaccine design [13], [14].

HIV-1 is characterized by an extraordinary genetic diversity, reflected by the presence of several clades (subtypes), a fact that represents a significant impediment to vaccine development. *Env* is the most variable HIV-1 gene, with up to 35% sequence diversity among clades, 20% diversity within clades, and 10% diversity in a single infected individual [15], [16], [17]. Several conserved epitopes have been defined by a small panel of neutralizing mAbs isolated using different experimental approaches. One epitope that appears to be relatively conserved and overlaps with the CD4 binding site (CD4bs) on the surface Env glycoprotein gp120 is recognized by mAb b12, which is the most potent and broadly-reactive mAb of such specificity [18], [19], [20]. This site was recently shown to be a significant target of neutralizing antibodies present in the sera of selected patients [21], [22]. However, b12 was derived from a phage library in which heavy and light chains have been randomly re-assorted, thus its relevance to naturally-occurring B cell responses in HIV-1 infection is unclear. A second epitope in a carbohydrate-rich region on the outer domain of gp120 is composed of glycans recognized by mAb 2G12, which shows an unusual interlocked VH domain-swapped dimer generating an extended and monovalent binding surface [23], [24], [25], [26]. The 2G12 epitope is not present in the majority of clade C isolates [27], but, of more concern, no 2G12-like activity has been detected in the sera of HIV-1 infected individuals [21], [22], suggesting that this type of neutralizing antibody may not be generally amenable to elicitation by B cells. A third target of the broadly-neutralizing mAb 4E10, and relatively broad mAbs 2F5 and Z13, is located on the membrane-proximal external region (MPER) of the transmembrane glycoprotein gp41 [28], [29], [30], [31]. As with 2G12, MPER-specific neutralizing antibodies are infrequently encountered in the sera of HIV-1-infected individuals [21], [32], [33], potentially reducing their relevance to vaccine development strategies. Moreover, MPER-specific mAbs have features that suggest they are products of autoreactive B cells, casting doubts over the possibility of inducing such antibodies by vaccination [34]. A fourth target of neutralizing mAbs is the V3 loop, which is immunogenic but also a highly variable region involved in Env-coreceptor binding. Antibodies to the V3 loop, such as mAbs 447-52D and F425-B4E8, are mostly effective against neutralization sensitive (Tier-1) viruses and have generally a limited breadth of reactivity almost exclusively specific for clade B viruses, reflecting their provenance from clade B-infected individuals [11], [35], [36], [37]. A cluster of human V3 mAbs raised from B cells derived from non-B clade-infected individuals showed variable neutralizing activity for two AG and two C clade primary isolates, although these were particularly neutralization sensitive [38]. Finally, the CD4-induced (CD4i) site has been described as a neutralization epitope primarily on Tier-1 isolates with limited accessibility on Tier-2 isolates [39]. Very recently, the isolation and partial characterization of two related MAbs from a clade A-infected donor that appear to have very broad and potent neutralization profiles was reported [40]. These MAbs recognize a discontinuous epitope only expressed on the native Env trimer, made up of segments of the CD4i, V2 and V3 loops. This publication is the first to provide new broadly neutralizing reagents from a non-clade B donor and provides proof-of-principle that new neutralization specificities remain to be discovered on HIV-1 Env.

Since the number of existing neutralizing mAbs with a relatively broad neutralization profile is very limited, and those described to date, with the exception of the Walker et al MAbs were isolated from clade B virus-infected donors, we undertook the task of isolating new human mAbs from HIV-1-infected donors. Our primary aims were: i) to isolate novel MAbs with cross-clade neutralizing activity from non-B clade donors; ii) to analyze the memory B cell repertoire in a representative panel of predominantly non-B clade-infected individuals. By using an improved memory B cell immortalization method [41], combined with high-throughput parallel screening with a panel of recombinant Env-based antigens, we isolated a panel of 58 human mAbs which we have characterized with regard to epitope specificity and breadth of neutralization. We have more fully characterized three mAbs from this panel that demonstrated complementary and relatively broad cross-clade neutralizing profiles that target the gp120 CD4bs and the V3 crown, and the gp41 heptad repeat 1 (HR-1).

## Materials and Methods

### Ethics Statement

The study was approved by the ethical committee at Institute of Tropical Medicine and Queen Mary University. All participants gave written informed consent.

### Cells and Reagents

TZM-bl and HOS.CD4-R5 cells were obtained from the NIH- AIDS Research and Reference Reagent Program (ARRRP). 293T/17 cells were obtained from the ATCC. MAbs 2G12 [29], 2F5, 4E10 [42], b12 [18], 3D6 [43] and 5F3 [29] were obtained from Polymun Scientific GmbH (Austria), as part of the Collaboration for AIDS Vaccine Discovery program.

### Viruses

The clade B and C HIV-1 Reference Panels of Env clones [44], [45] and the SF162 clone were obtained through the NIH-ARRRP. Other non-clade B isolates were provided by the Comprehensive Antibody Vaccine Immune Monitoring Consortium (CA-VIMC). HIV-1 subtype B clone JRFL was provided by Dennis Burton (Scripps Institute, La Jolla, US). Pseudoviruses were produced by co-transfecting HEK293T/17 cells with the *env*-expressing plasmids and the complementing viral-genome reporter gene vector, pNL4-3.Luc^+^.E^−^R^+^ (kindly provided by John R. Mascola, VRC, NIAID, NIH, US).

### Recombinant HIV-1 Envelope Glycoproteins

Recombinant gp140 from HIV-1 isolates 92UG37, CA18, CN54, k530, UG21 and BR29 were provided by Simon Jeffs (Imperial College London, UK). Recombinant gp120 from HIV-1 isolates CM235, 93TH975, BaL and SF162 were obtained through the NIH-ARRRP, while gp160 from isolates MN and LAI were purchased by Prospec-Tany Technogene LTD (Israel). Recombinant YU2 gp120 and the two mutants D368R and I420R were provided by John R. Mascola and Richard Wyatt (VRC, NIAID, NIH, US). Recombinant soluble CD4 and recombinant gp120 from HIV-1 IIIB and CN54 were obtained from the CFAR, NIBSC (UK). The recombinant ectodomain of gp41 from isolate HxB2 (amino acids 541-682) was purchased by Vybion Inc. (US). HR-1 is a modified version of gp41 [46] exposing residues HLLQLTVWGIKQLQARILAVE (HxB2). 5HB was produced as described [47]. HR-1-FP comprises gp41-residues 512-594 (HxB2) including the fusion peptide and HR-1. HR-2 (HxB2) comprises gp41 residues 591–693 (HxB2) including the cys-loop region, HR-2 and MPER flanked by two coiled coil regions.

### Patient Selection and MAb Isolation

Patients were selected for inclusion into the study on the basis of the clade of their infecting virus (predominantly non-B clade) and on the ability of their plasma to neutralize a panel of HIV-1 primary isolates using two differerent assays. At the Institute for Tropical Medicine a panel of 4 primary A strains (VI191, 92UG37, VI820, VI 1031), 4 primary C strains (VI829, VI882, VI1144, VI1358) and 6 primary CRF02 strains (VI1090, VI2680, CI20, CA18, VI1380, VI2727) was used to select patients with >80% neutralization was measured at a 1/20 dilution of plasma. The plasma dilution was mixed with virus for 24 hours and absorbed to PHA/IL-2 stimulated freshly isolated PBMC for 1 hour. Virus-antibody mixture was then removed by extensive washing and p24 ELISA was performed after 14 days of culture to determine neutralization. At Queen Mary University of London, the TZMbl-based recombinant pseudovirus assay was employed with a selected panel of Tier 2-type clade A, B, C, CRF02_AG, _AE, F and BF viruses to measure >50% neutralization at a 1/20 dilution. MAbs were isolated using a previously described improved EBV immortalization method [41]. Culture supernatants were harvested 14 days after immortalization and assayed in parallel for binding to trimeric gp140 proteins (UG37, clade A and CN54, clade CRF07_BC) [48], monomeric gp120 (CN54, CRF07_BC and IIIB, clade B) and gp41 recombinant ectodomain (HxB2, clade B). Those that were positive were then subcloned and grown up on a large scale for supernatant purification. The purity of all mAb preparations was assessed using a chromogenic LAL endotoxin assay (Genscript) and endotoxin levels were always <0.05 EU/ml.

### Binding Assays

A standard ELISA was used to determine binding of mAbs to the panel of HIV-1 Envs. Briefly, ELISA plates were coated with each Env antigen, blocked with 10% FCS in PBS, incubated with human mAbs and washed. Bound mAbs were detected by incubation with AP-conjugated goat anti-human IgG (Southern Biotech). Plates were then washed, substrate (p-NPP, Sigma) was added and plates were read at 405 nm. The relative affinities of mAbs binding to respective coated antigens were determined with ELISA by measuring the concentration of each mAb required to achieve 50% maximal binding at saturation (K_50_). The ability of mAbs to inhibit binding of sCD4 to gp120 or gp140 was evaluated by ELISA. Serial dilutions of mAbs were pre-incubated with gp120 (or gp140) and added to plates pre-coated with sCD4. After 1 h plates were washed and incubated with sheep polyclonal antibody D7324 (Aalto Bio-Reagents) followed by washing, incubation with AP-conjugated rabbit anti-sheep IgG antibody (Abcam, Cambridge, UK), extensive washing and detection as above.

### Competition Assay

MAbs were purified on Protein G columns (GE Healthcare) and biotinylated using the EZ-Link NHS-PEO solid phase biotinylation kit (Pierce). The competition between unlabeled and biotinylated mAbs for binding to immobilized Env antigens was measured by ELISA. Briefly, unlabelled competitor mAbs were added at different concentrations. After 1 h biotinylated mAbs were added at a concentration corresponding to the 70–80% of the maximal OD level. After incubation for 1 h, plates were washed and bound biotinylated mAb was detected using AP-labeled streptavidin (Jackson Immunoresearch). The percentage of inhibition was tested in triplicates and calculated as follow: (1−[(ODsample-ODneg ctr)/(ODpos ctr - ODneg ctr)])×100.

### Peptide Scanning

Overlapping linear 15-mer and cyclized 15-mer peptides based on gp160 of HIV-1 UG037 and 93MW965 and the overlapping 15-mer peptides of gp41 strain SF162 were synthesized on polypropylene support (minicards), and were tested for reactivity with mAbs as described [49], [50]. Pepscan peptide binding analysis was carried out by an ELISA-based format in which the colored substrate was quantified with a charge-coupled device (CCD) - camera and an image processing system. The values mostly ranged from 0 to 3000, a log scale similar to 1 to 3 of a standard 96-well plate ELISA-reader.

### Neutralization Assays

A single-cycle infectivity assay was used to measure the neutralization of luciferase-encoding virions pseudotyped with the desired HIV-1 Env proteins, as previously described [51]. Briefly, appropriate dilutions of the virion-containing culture supernatants were pre-incubated at 37°C for 1 h with mAbs at various concentrations. The virus-mAb mixtures were added to HOS-CD4-CCR5 cells and incubated for 3 days at 37°C. A similar protocol was used for supernatants screening using TZM-bl cells [45]. The cells were then lysed with Britelite reagent (Perkin-Elmer) and the relative light units in the cell lysates were determined on a luminometer microplate reader (Veritas, Turner Biosystems). The 50% inhibitory dose (IC50) was defined as the sample concentration at which relative luminescence units were reduced 50% compared to virus control wells. Some of the neutralization data for the reference mAbs b12, 2G12, 2F5 and 4E10 are taken from previous analyses [41], [42] carried out under identical standardized conditions to those used for the new mAbs reported here. A multi-cycle virus replication assay was used to measure the neutralization of replication competent luciferase-encoding viruses in 5.25.EGFP.Luc.M7 cells [52]. This cell line is a genetically engineered clone of CEMx174 that expresses multiple SIV and HIV-1 entry receptors (CD4, CCR5, CXCR4, GPR15/Bob) [53]. For the neutralization assay, 5000 tissue culture infectious dose 50 (TCID_50_) of virus was incubated with multiple dilutions of test sample in triplicate for 1 hr at 37°C in a total volume of 150 µl in 96-well flat-bottom culture plates. A 100 µl suspension of cells (5×10^5^ cells/ml of growth medium containing 25 µg DEAE dextran/ml) was added to each well. Plates were incubated until approximately 10% of cells in virus control wells were positive for GFP expression by fluorescence microscopy (approximately 3 days). At this time, 100 µl of cell suspension was transferred to a 96-well white solid plate (Costar) for measurements of luminescence using the Britelite Luminescence Reporter Gene Assay System (PerkinElmer Life Sciences).

## Results

### Isolation of HIV-1 Neutralizing mAbs from Memory B Cells of Infected Donors

Plasma samples were obtained from patients entered into the Antwerp and London cohorts who were infected with a variety of HIV-1 clades. Patients were selected for MAb preparation on the basis of the clade of their infecting virus (predominantly non-B clade), and on the ability of their plasma to neutralize, to >80% at a 1/20 dilution, members of a selected panel of Tier 2-type clade A, B, C, CRF02_AG, AE, F and BF viruses in both a PBMC-based infectious primary isolate virus assay [54] and in the TZMbl-based recombinant pseudovirus assay [55]. In some cases only a restricted plasma neutralization analysis was possible due to limiting amounts of patient plasma. Twelve and nine infected donors from the London and Antwerp cohorts respectively were selected for B cell immortalization. Summarized clinical and virological data including HIV-1 clade, viremia, CD4^+^ T cell counts, therapy and breadth of neutralization are shown in **Table S1**.

IgG^+^ memory B cells were isolated from the selected donors and immortalized with EBV in the presence of CpG as described [41]. The immortalization efficiency of memory B cells from HIV-1 patients was significantly lower than that of non-HIV-1-infected donors (3% versus 20%, n = 21, p<0.001). This finding may be explained by the recent report that HIV-specific B cells are present within a population of “exhausted” memory B cells, characterized by the expression of inhibitory receptors and low levels of CD21 [56], [57]. To maximize detection of antibodies recognizing conserved features, cell culture supernatants were screened by parallel high-throughput ELISAs using five recombinant HIV-1 Env proteins, including trimeric gp140 [48], monomeric gp120 and gp41 recombinant ectodomains. From the 21 donors interrogated we selected 58 B cell clones producing mAbs that bound to at least one of the screening antigens. The mAbs were purified and further characterized for binding specificity and neutralizing activity using an extended panel of recombinant Env proteins and pseudoviruses representative of several HIV-1 clades with diverse coreceptor usage, geographic origin and conformation.

The binding data for each mAb are displayed in **Figure S1** and expressed as half-maximal binding concentrations at equilibrium (K_50_), which approximate to the equilibrium dissociation constants [58]. Of the 58 mAbs, 37 bound to gp120 and 21 to gp41. Several gp120-specific and gp41-specific mAbs showed a broad pattern of reactivity with most recombinant proteins, although usually within a broad range of K_50_ values. Overall, there was no relationship between the donor's HIV-1 clade and the clade specificity of the isolated mAbs.

**Figure 1:**
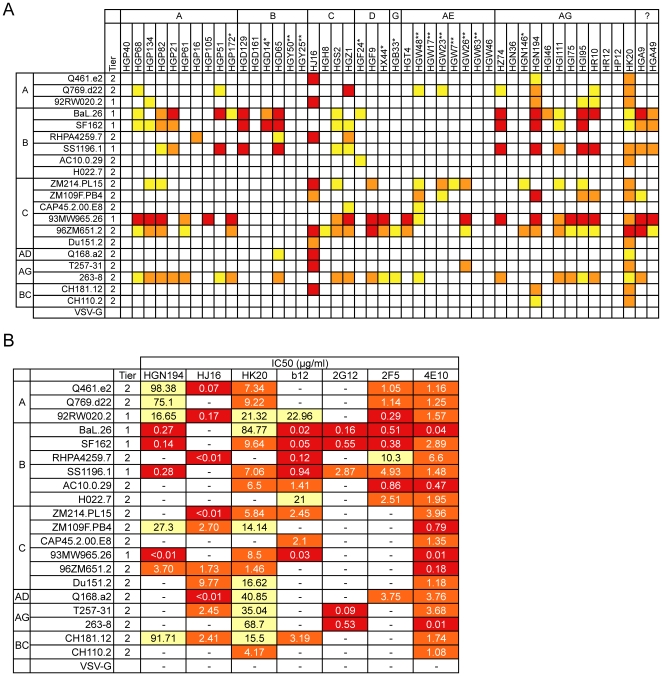
Neutralization of 20 HIV-1 isolates in HOS.CD4-R5 cells by a panel of human mAbs. (A) 46 mAbs (upper rows) were purified and tested in triplicates at a fixed concentration (100 µg/ml; *50 µg/ml and **25 µg/ml) for their capacity to neutralize 20 HIV-1 pseudoviruses (left columns) representing 6 different clades and both Tier-1 and Tier-2 isolates using HOS.CD4-R5 as target cells. Indicated is also the donor's HIV-1 clade. White, neutralization below 50%; yellow, 51–69%; orange, 70–89% and red, 90–100% neutralization. VSV-G pseudotyped HIV-1 was also tested as a negative control. (B) HK20, HGN194 and HJ16 were tested in parallel with b12, 2G12, 2F5 and 4E10 for their capacity to neutralize 20 HIV-1 pseudoviruses representing 6 different clades and both Tier-1 and Tier-2 isolates using HOS-CD4.R5 cells as in (A). Shown are IC50 values in µg/ml. -, indicates IC50 values >100 µg/ml.

The neutralizing activity of the mAbs was initially measured using 20 pseudotyped HIV-1 primary isolate *envs* characterized by different sensitivity to neutralization [44], [45]. The mAbs were tested at a fixed concentration (100 µg/ml) in a luciferase-based neutralization assay using HOS-CD4.R5 as target cells. Out of the 46 mAbs tested against the whole virus panel, 37 showed neutralizing activity on at least one isolate (**Figure 1A**). Three mAbs, HJ16, HGN194 and HK20, stood out for their breadth of neutralizing activity, neutralizing 10, 11 and 17 out of the 20 pseudoviruses, respectively (**Figure 1A**). These mAbs were further characterized in the same assay for their potency (**Figure 1B**). HJ16 showed high neutralizing activity, while HGN194 and HK20 were less potent. In addition, while most antibodies preferentially neutralized Tier-1 isolates, HJ16 preferentially neutralized Tier-2 isolates (**Figure 1A-B**).

Since it has been reported that HIV-1 neutralization is target cell type-sensitive [59], [60], we next used TZM-bl as target cells and titrated the mAbs against a larger panel (n = 46) of predominantly (38/46) Tier-2 pseudoviruses (**Table S2**). Generally the results obtained with the extended panel confirmed the previous results and showed that several mAbs had potent neutralizing activity, with IC_50_ values <20 ng/ml. A striking exception was HK20, which neutralized 17/20 pseudoviruses in the HOS-based assay, but only 3/46 pseudoviruses in the TZM-bl based assay.

**Figure 2:**
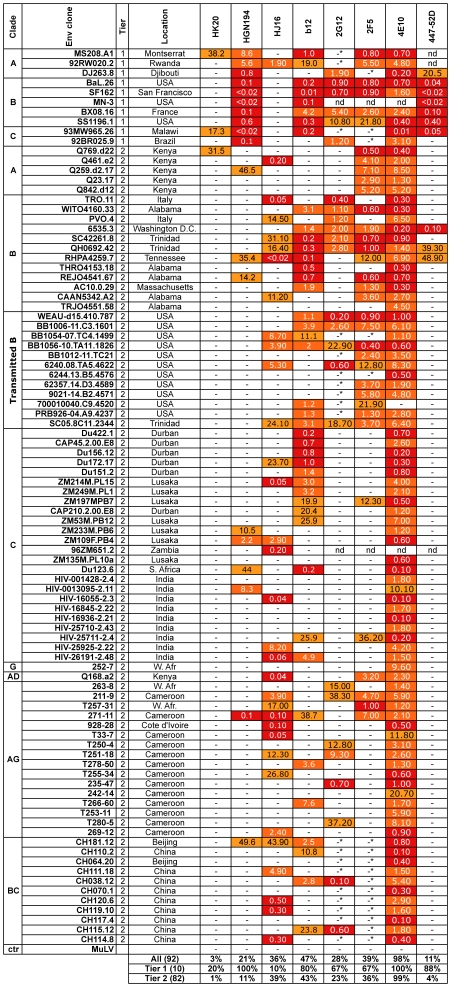
Neutralization of 92 HIV-1 isolates in TZM-bl cells by HK20, HGN194, HJ16. HK20, HGN194 and HJ16 were tested in parallel with b12, 2G12, 2F5, 4E10 and 447-52D for their capacity to neutralize 92 HIV-1 pseudoviruses representing 7 different clades and both Tier-1 and Tier-2 isolates using TZM-bl as target cells. Shown are IC50 values in µg/ml. -, indicates IC50 values >50 µg/ml, -* mAb tested starting from 25 µg/ml, nd, not determined. MuLV pseudotyped HIV-1 was also tested as a negative control.

We established the breadth of neutralization of these three novel mAbs by comparing them in the TZMbl assay with the five mAbs currently considered to be the most broadly reactive, namely b12, 2G12, 2F5, 4E10 and 447-52D. We used a large multi-clade panel comprising 10 Tier-1 and 82 Tier-2 isolates including clade B early-transmitted viruses [61]. The three new mAbs showed a pattern that was clearly distinct from that of previously described mAbs (**Figure 2**). In particular, HGN194 neutralized with high potency all Tier-1 viruses from clade A, B and C and 11% of Tier-2 viruses. By contrast HJ16 neutralized only 1 out of 10 Tier-1 viruses, but neutralized 39% of Tier-2 viruses. Thus HJ16 is comparable to b12 and 2F5 in terms of percentage of Tier-2 isolate neutralization and is superior to 2G12 and 447-52D. 4E10, as previously reported [27], [41] showed an extremely broad pattern of reactivity. Taken together the above results indicate that novel neutralizing mAbs can be isolated from memory B cells of non-clade B HIV-1-infected individuals, some of which display relatively broad neutralizing activity. Since one of our primary aims was to isolate and characterize novel MAbs with unusually broad and potent neutralization activity, we focused our attention on HJ16, HK20 and HGN194.

### HJ16, a Neutralizing mAb Binding a Novel Epitope Proximal to the CD4bs

**Figure 3:**
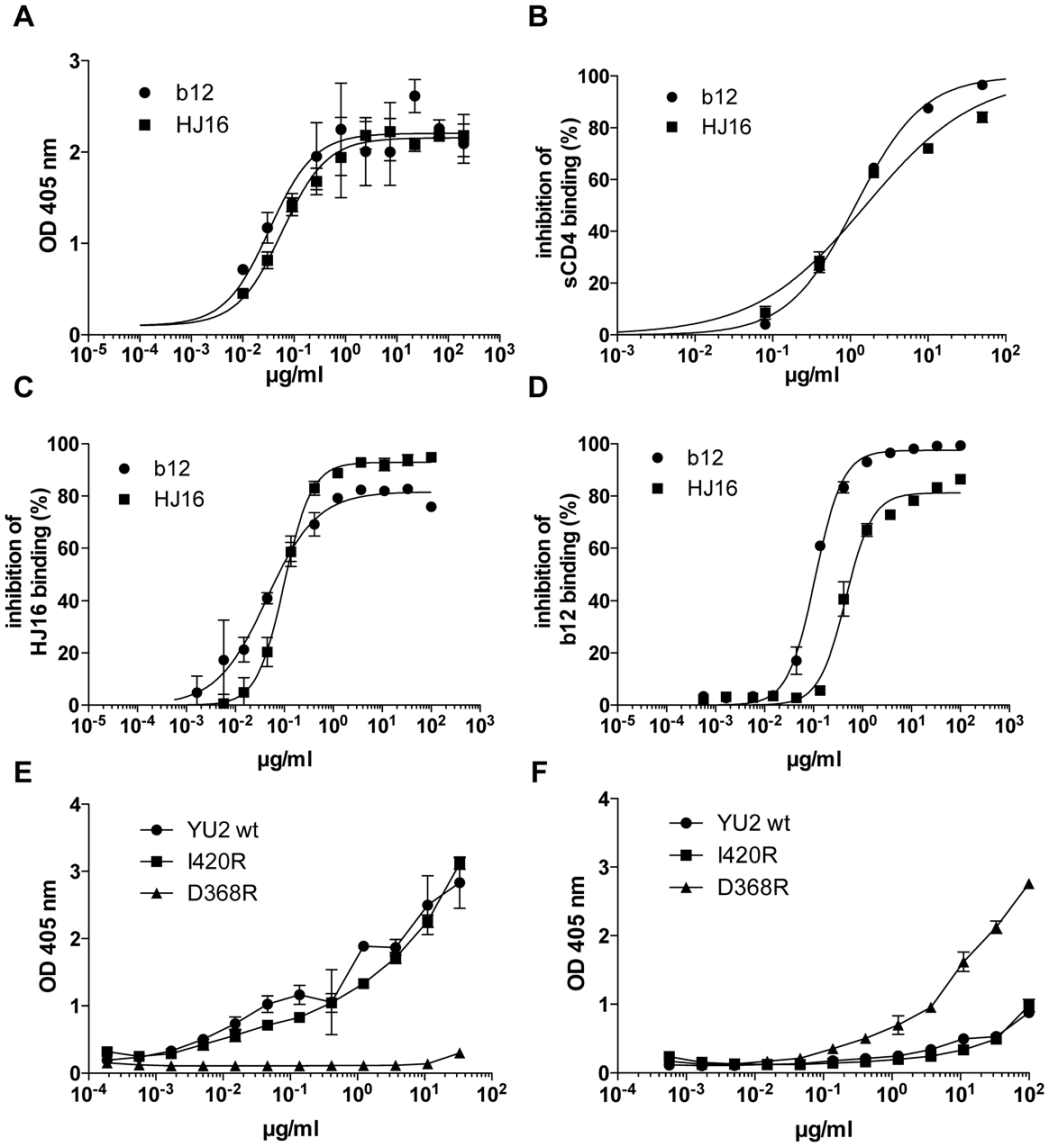
HJ16 binds to a CD4bs epitope distinct from that recognized by b12. (A) Binding of HJ16 and b12 to IIIB gp120 envelope protein. (B) Inhibition of IIIB gp120 binding to immobilized sCD4 by HJ16 and b12. (C–D) Inhibition of binding of HJ16 (C) or b12 (D) to immobilized gp120 by increasing concentrations of unlabeled HJ16 or b12. (E–F) Binding of b12 (E) or HJ16 (F) to YU2 wt gp120 and to the CD4i (I420R) or CD4bs (D368R) mutant YU2 proteins. Shown is mean ± SD of triplicates.

MAb HJ16, derived from a donor infected with clade C, has a unique neutralization profile with potent and selective neutralization of multiple Tier-2 pseudoviruses (**Figure 1B** **and** **Figure 2**). When compared with the CD4bs-specific mAb b12 for gp120 binding, HJ16 showed similar binding curves (**Figure 3A**) and inhibited to a comparable extent the binding of gp120 to solid-phase sCD4 (**Figure 3B**, IC_50_ values of 1.57 and 1.16 µg/ml, respectively). However, cross-competition analysis of HJ16 and b12 for binding to gp120 revealed incomplete heterologous inhibition, with plateau values of approximately 80% (**Figure 3C-D**). These results suggest that HJ16 and b12 recognize related but non-overlapping CD4bs-proximal epitopes. To further characterize HJ16 specificity we measured its binding to two YU2 gp120 mutants: the D368R mutant, which is not bound by CD4 or CD4bs-specific mAbs [21] and the I420R mutant, which is not recognized by CD4i-specific mAbs [62]. As already reported, b12 bound the I420R CD4i mutant, but failed to recognize the D368R CD4bs mutant. By contrast, HJ16 bound both mutants and indeed bound better to the D368R CD4bs mutant compared to the wild-type molecule (**Figure 3E-F**). Attempts to map the epitope by Pepscan analysis using overlapping linear peptides were unsuccessful (not shown), consistent with HJ16 recognition of a discontinuous epitope.

HJ16 and b12 neutralized 1/10 and 8/10 Tier-1 and 32/82 and 35/82 Tier-2 pseudoviruses, respectively (**Figure 2**). Interestingly, 22/32 Tier-2 isolates neutralized by HJ16 were not neutralized by b12, and reciprocally 24/35 Tier-2 isolates neutralized by b12 were not neutralized by HJ16. Another interesting finding is that HJ16 does not discriminate between clades as much as b12, 2G12 and 2F5 (e.g. b12 and 2G12 rarely neutralize clade A isolates while 2F5 and 2G12 rarely neutralize clade C isolates, **Table S3**). These results reveal a largely non-overlapping pattern of reactivity of HJ16 and b12 and suggest that the combination of these two mAbs could be effective against a dominant fraction of HIV-1 isolates (57/82, i.e. 69%). Taken together these results are consistent with HJ16 recognition of a surface proximal to the CD4bs domain within gp120, but with an epitope completely distinct from that recognized by b12.

### HGN194, a Broadly Neutralizing mAb That Binds to the V3 Crown

**Figure 4:**
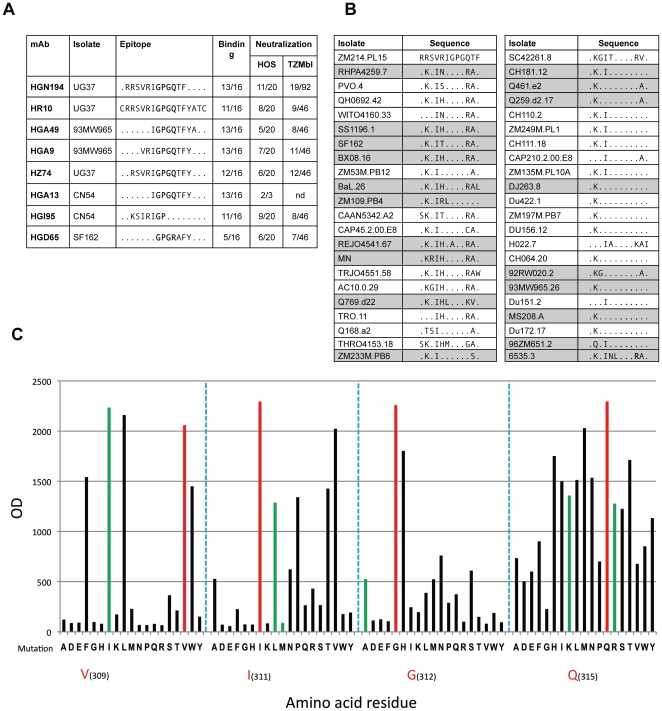
Epitope mapping of V3-specific mAbs by linear and circular peptide scanning. (A) Eight gp120-specific mAbs were mapped with linear and cyclic peptides to the V3 region. Shown is the HIV-1 isolate used for the mapping, the minimal epitope, the binding breadth expressed as number of recombinant Env proteins out of the 16 tested, and the fraction of isolates neutralized in the HOS and TZMbl-based neutralization assays, respectively. (B) Alignment of the region corresponding to the epitope recognized by mAb HGN194 in 44 HIV-1 isolates. Co-receptor binding residues are bold. Highlighted in grey are the isolates neutralized by HGN194 either in the TZMbl-based or in the HOS-based assays. (C) Replacement analysis at positions R(307), S(308), V(309), R(310), I(311), G(312), Q(315), T(316 and F(317) of the epitope recognized by HGN194. Shown is the binding to 92 peptides carrying various amino acid substitutions at positions critical for maintenance of MAb binding. The bars represent ELISA values of HGN194 binding with the wt peptide (red) and the variant peptide (black). The amino acid replacements corresponding to V3 sequences from all isolates shown in (B) are highlighted with green bars. The binding of antibody to each peptide was tested in a PEPSCAN-based ELISA. Numbering according to HIV-1 HXB2.

MAb HGN194, isolated from a donor infected with CRF02_AG clade, neutralized 11/20 pseudoviruses in the HOS-based assay and 19/92 pseudoviruses in the TZM-bl-based assay. Of note, HGN194 neutralized all Tier-1 isolates tested (10/10 neutralized) across clades A, B, C and recombinant AG and BC. Using Pepscan analysis with linear and cyclic peptide libraries of gp120 the epitope recognized by HGN194 was mapped to the sequence RRSVRIGPGQTF in the crown of the V3-loop (**Figure 4A**). Similarly, the epitope of 7 additional gp120-specific mAbs was mapped to the same V3 region using different peptide libraries generated from the sequence of the isolate to which each mAb was mostly reactive (**Figure 4A**). The minimal sequence recognized by these mAbs ranged from 7 to 17 amino acids and, with a single exception, comprised the G(A)PGR/Q/K sequence, which is modeled to interact with CCR5 or CXCR4 during the viral entry process [63]. However, in contrast to HGN194 which neutralizes with high potency all Tier-1 isolates tested, the other V3-specific mAbs neutralized only a few Tier-1 isolates (**Figure 1A-B** **and** **Figure 2**), similar to the activity of other recently characterized V3 loop-specific mAbs derived from non-B clade infected individuals [38]. We confirmed the neutralization activity of HGN194 in an M7 cell-based assay challenged with replication-competent HIV-1 viruses carrying a Luc reporter [52] and we observed that 3/5 clade B viruses were neutralized (Table S5).

To better characterize the epitope recognized by HGN194 we performed a replacement scanning of each position with the 18 complementary amino acids. This analysis revealed only 3 positions (RRSVRIGPGQTF) where amino acid substitutions abrogated binding. Remarkably, only one mutation out of the 21 found in viral isolates (I to M in position 6) affected binding of HGN194 (**Figure 4B–C**). However, we observed that several HIV-1 isolates that were not neutralized by HGN194 encoded the same amino acid sequence shared by other HIV-1 isolates that were neutralized (**Figure 4B**). For instance, the epitope RKSVRIGPGQTF on 93MW965.26, which is strongly neutralized by HGN194 (i.e. IC50 <0.02 µg/ml), is shared with Du422.1, ZM197M.PB7 and Du172.17, isolates not neutralized by HGN194. This implies that the HGN194 V3 epitope is unavailable for antibody recognition on the assembled trimer of these non-neutralized isolates.

Taken together these results indicate that HGN194 has unusual potency and breadth for a V3-specific monoclonal antibody. Of note, HGN194 appears to have a broader reactivity than 447-52D since it neutralizes all Tier-1 isolates and 11% of Tier-2 isolates, while 447-52D neutralizes 88% of Tier-1 and 4% of Tier-2 isolates (**Figure 2** and **Table S3**). Another V3 mAb with unusual breadth of activity is F425-B4e8 [37]. We have not compared HGN194 with F425 here, but F425-B4e8 appears somewhat broader than 447-52D, neutralizing 1 clade C and 2 clade D pseudoviruses [37]. Further comparison between F425-B4e8 and HGN194 is difficult as different viruses and assays were used to determine neutralization breadth and potency.

### HK20, an HR-1 Specific mAb with Target Cell-Specific Neutralizing Activity

**Figure 5:**
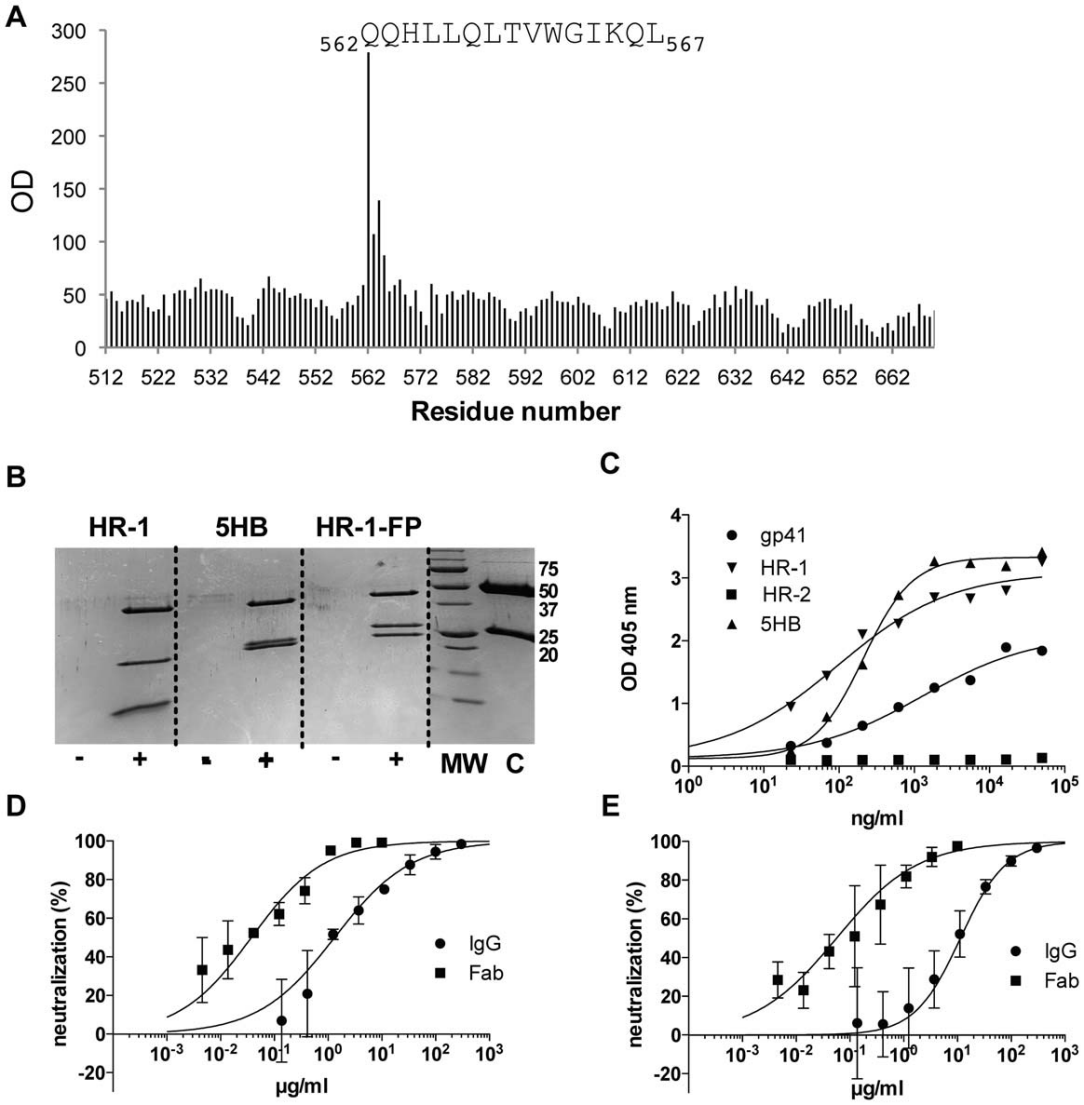
HK20 binds to the HR-1 region within gp41. (A) HK20 binding at 4 ug/ml to all overlapping linear peptides (15-mer peptides overlapping by 14 residues) spanning the gp41 sequence of the HXB2 isolate. Numbers at X-axis denote the first amino-terminal residue of the 15-mer gp41 peptide (numbering according to HIV-1 HXB2 sequence). Y- axis similar to Figure 4C (B) Immunoprecipitation of HR-1, 5HB and HR-1-FP constructs in the presence (+) or absence (−) of HK20 mAb. Proteins were separated in 10% polyacrylamide gels under reducing conditions and stained with Coomassie blue. C, HK20 mAb alone; MW, molecular weight. (C) HK20 binding to gp41 constructs by ELISA. (D–E) Neutralization of 96ZM651.2 (D) and CH064.20 (E) HIV-1 primary isolates by HK20 IgG and Fab fragments in a HOS-based assay.

HK20 was initially characterized as a gp41-specific mAb with broad neutralizing activity in the HOS-based assay but lacking robust activity in the TZM-bl assay. To map the epitope, we tested HK20 against all overlapping 15-mer peptides of the extracellular region of HIV-1 gp41. HK20 bound peptide QQHLLQLTVWGIKQL that overlaps the hydrophobic pocket sequence of HR-1 (**Figure 5A**). The specificity of HK20 was confirmed by immunoprecipitation of the 5-helix bundle (5HB) construct [47] and of a trimeric HR-1 construct that includes the gp41 fusion peptide, indicating that the fusion peptide does not interfere with HK20 binding (**Figure 5B**). In ELISA, HK20 bound to 5HB and HR-1 gp41 constructs with K_50_ values of 210 and 95 ng/ml, respectively and also to the gp41 ectodomain, although with lower avidity (1.27 µg/ml) (**Figure 5C**), a finding that may be due to the partial unfolding of solid phase-bound gp41. Taken together, the above results indicate that HK20 recognizes a highly conserved site within the HR-1 region of gp41.

Since HR-1 occupies a restricted surface only transiently exposed during the HIV-1 entry process, we hypothesized that accessibility of the HK20 target epitope might be limited by the size of an IgG molecule. We therefore compared intact IgG and Fab fragments of HK20 for their capacity to neutralize a panel of 22 pseudoviruses in the HOS-based assay. The HK20 Fab fragments showed markedly increased breadth and potency compared to IgG, being able to neutralize 21/22 isolates with IC_50_ values ranging from 0.01 to 5.7 µg/ml (**Figure 5D-E** **and Table S4**). By contrast, HK20 IgG neutralized 19/22 isolates with IC_50_ values ranging from 1.46 to 84 µg/ml. In addition, when the HK20 Fab fragment was tested in the TZM-bl assay it neutralized 10 out of 33 isolates with IC_50_ values ranging between 0.4 and 8.8 µg/ml (**Table S2**), thus suggesting that the lack of activity observed with HK20 IgG was possibly associated to a different susceptibility of this cell line to this type of entry inhibitors. Overall, these findings suggest that the reduced size of the Fab molecule allows increased access to the HR-1 region and imply that HR-1 accessibility is largely dependent on the target cell used. This is similar in concept to the enhanced neutralization profiles of Fab and ScFv specific for the CD4i surface [39]. Further studies using M7 cells [52] challenged with replication-competent HIV-1 viruses carrying a Luc reporter showed that HK20 exhibits broad neutralization consistent with the results from the HOS cell assay but unlike the TZM-bl assay, being able to neutralize 4 out 5 viruses tested (**Table S5**).

### Broad Coverage of gp41 and gp120 Epitopes

**Figure 6:**
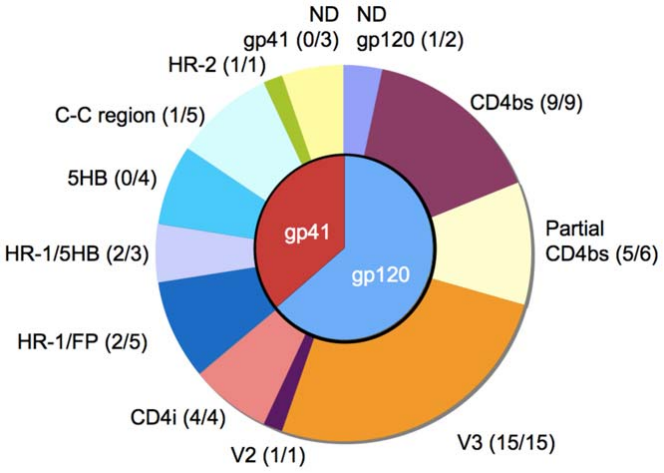
Epitope specificity and neutralizing activity of the mAb panel. The pie chart shows the specificity of the 58 mAbs isolated from the 21 interrogated individuals. The fraction of antibodies with neutralizing activity against at least one isolate is indicated in parentheses. Partial CD4bs, incomplete inhibition of CD4 binding; HR-1/FP, epitope located in between HR-1 and the fusion peptide as determined by 5F3 competition; HR-1/5HB, recognition of trimeric HR-1 and 5-helix bundle; 5HB, 5 helix bundle; C-C region, gp41 immunodominant region; HR-2, heptad-repeat 2; ND, not defined epitopes within gp120 or gp41.

The epitopes recognized by the 55 remaining mAbs were mapped using three experimental approaches: i) cross-competition against mAbs of known specificity or soluble CD4; ii) binding to gp120 mutants or gp41 constructs representing different conformational intermediates; iii) Pepscan analysis. The results of the analysis performed on the gp120-specific and gp41-specific mAbs are presented in **Table 1** and **Table 2**, and the data are summarized in a pie chart in **Figure 6**.

**Table 1:** Epitope mapping of gp-120-specific mAbs.

	Cross-competition with:						Binding to:				Neutralization	
mAbs	HR10	HGA9	HJ16	b12	2G12	sCD4	YU2 wt	YU2 I420R	YU2 D368R	Specificity	HOS	TZM-bl
HGF9	+	+/−	−	−	−	−	−	−	−	V3	4/20	6/46
HGT4	+	+/−	−	−	−	−	−	−	−	V3	2/20	2/36
HGA13	+	+/−	−	−	−	−	+	+	+	V3	2/3	nd
HGA49	+	+/−	−	−	−	−	+	+	+	V3	5/20	8/46
HGA9	+	+	−	−	−	−	+	+	+	V3	7/20	11/46
HGD129	x	+	−	−	−	−	+	+	+	V3	3/20	7/46
HGD65	x	+	x	x	x	−	+	+	+	V3	6/20	7/46
HGI95	+	+	−	−	−	−	+	+	+	V3	9/20	8/46
HGN194	+	+	−	−	−	−	+	+	+	V3	11/20	19/92
HGP21	x	+	x	x	x	−	+	+	+	V3	4/20	5/46
HGP51	x	+	x	x	x	−	+	+	+	V3	4/20	5/46
HGW48	−	+	−	−	−	−	+	+	+	V3	6/20	nd
HR10	+	+	−	−	−	−	+	+	+	V3	8/20	9/46
HZ74	+	+	−	−	−	−	+	+	+	V3	6/20	12/46
HGP27	−	+	−	−	−	−	+	+/−	+	V3	1/3	nd
HX44	−	−	−	x	x	−	+	−	+	CD4i	3/20	2/46
HGP105	−	−	−	x	x	−	+	−	+	CD4i	1/20	1/46
HGW7	−	−	−	x	x	−	+	−	+	CD4i	1/20	nd
HGY38	−	−	−	x	x	−	+	−	+	CD4i	2/3	nd
HGD14	x	−	x	+	−	+/−	+	−	−	CD4bs∼	2/20	5/46
HR15	−	−	−	x	−	+/−	−	−	−	CD4bs∼	1/3	nd
HGF12	−	−	−	−	−	+/−	+	+	+	CD4bs∼	0/3	0/46
HGI46	x	−	x	x	−	+/−	+	+/−	+	CD4bs∼	1/20	5/46
HGI75	−	−	+/−	x	−	+/−	+	+/−	−	CD4bs∼	3/20	2/46
HGP172	−	−	+/−	+	−	+/−	+	+/−	−	CD4bs∼	4/20	6/46
HGP134	−	−	+/−	x	−	+	+/−	−	−	CD4bs	6/20	4/46
HGI111	−	−	+/−	+	−	+	+	+/−	−	CD4bs	7/20	4/46
HGP31	−	−	x	+/−	−	+	+	+/−	−	CD4bs	2/3	5/45
HGP61	−	−	+/−	x	−	+	+	+/−	−	CD4bs	3/20	8/46
HGP82	−	−	+	+	−	+	+	+/−	−	CD4bs	7/20	8/46
HGS2	−	−	−	+	−	+	+	+/−	+/−	CD4bs	7/20	4/46
HGW26	−	−	−	+	−	+	+	+/−	+/−	CD4bs	3/20	nd
HGZ1	−	−	+/−	+	−	+	+	+/−	+/−	CD4bs	8/20	10/46
HJ16	x	−	+	x	−	+	+/−	+/−	+	CD4bs	10/20	33/92
HGP68	−	−	−	x	−	−	+	+/−	+	V2	6/20	4/46
HR12	−	−	−	x	−	−	−	−	−	?	0/20	2/46
HP12	x	−	−	x	−	−	−	−	−	?	0/20	0/46
b12	−	−	+/−	+	−	+	+	+	−	CD4bs	11/20	43/92

Cross competition with biotinylated mAbs of known specificity or sCD4: +, 90–100% inhibition, +/−, 50–90% inhibition, −, <50% inhibition. The x indicates that it was not feasible to evaluate the competition due to the lack of binding to the immobilized gp120 protein. Binding by ELISA to YU2 gp120 wt and mutants in the CD4i and the CD4bs. Specificity assignment (CD4bs∼ indicates partial inhibition of sCD4 binding). Shown is also the number of isolates neutralized in the HOS-based and TZM-bl based assays.

**Table 2:** Epitope mapping of gp41-specific mAbs.

	Cross-competition with:					Binding to:			Epitope	Specificity	Neutralization	
mAbs	2F5	4E10	3D6	5F3	HK20	HR1	5HB	HR2			HOS	TZM-bl
HGN158	−	−	−	−	−	−	+	−		5HB	0/3	nd
HGN36	−	−	−	−	−	−	+	−		5HB	0/20	0/46
HGW34	−	−	−	−	+	+/−	+	−		5HB	0/3	nd
HGW46	−	−	−	−	−	−	+	−		5HB	0/20	0/46
HGK129	−	−	+	−	−	−	−	+	LLGIWGCSGKLIC	C-C	0/3	nd
HGN146	−	−	+	−	−	−	−	+	LLGIWGCSGKLIC	C-C	2/20	nd
HGN35	−	−	+	−	−	−	−	+	SGKLIC	C-C	0/3	nd
HGW17	−	−	+	−	−	−	−	−		C-C	0/20	nd
HGY25	−	−	+	−	−	−	−	−		C-C	0/20	nd
HGP40	−	−	−	−	−	−	−	−		gp41 only	0/20	0/46
HGP48	−	−	−	−	−	−	−	−		gp41 only	0/3	nd
HGY50	−	−	−	−	−	−	−	−		gp41 only	0/20	0/46
HK20	−	−	−	−	+	+	+	−	QQHLLQLTVWGIKQL	HR-1	17/20	3/92
HGB33	−	−	−	−	+	−	+	−		HR1/5HB	2/20	0/46
HGW63	−	−	−	+	+	+	+	−		HR1/5HB	0/20	nd
HGD161	−	−	−	+	+	+	+	−		HR-1/FP	0/20	0/46
HGP16	−	−	−	+	+	+	+	+/−		HR-1/FP	1/20	1/46
HGW23	−	−	−	+/−	+/−	+	+	−		HR-1/FP	3/20	nd
HGN91	−	−	−	+/−	+/−	+	+	−		HR-1/FP	0/3	nd
HGH8	−	−	−	+/−	+/−	−	+	−		HR-1/FP	0/20	0/46
HGF24	−	−	−	−	+/−	−	+	+	TNLIYTLIEESQN	HR-2	2/20	4/46
3D6	−	−	+	−	−	−	−	+/−	GCSGKLICTTAVPW	C-C	nd	nd
5F3	−	−	−	+	−	+	+	−	STMGAASITLTAQARQ	FP	nd	nd
2F5	+/−	+/−	−	−	−	−	−	+/−	DKW	MPER	10/20	35/90
4E10	+	+	−	−	−	−	−	+/−	WFDI	MPER	20/20	89/90

Cross competition with biotinylated mAbs of known specificity: +, 90–100% inhibition, +/−, 50–90% inhibition, −, <50% inhibition. Binding to different gp41 constructs by ELISA. Minimal linear epitopes using the Pepscan analysis and specificity assignment. C-C, C-C loop; 5HB, 5-helix bundle; FP, fusion peptideShown is also the number of isolates neutralized in the HOS-based and TZM-bl based assays.

Thirty-seven mAbs bound to gp120 targeting primarily the CD4bs and the V3 loop and to a lesser extent the CD4i site (**Table 1**). A first group of mAbs was assigned to the V3 loop based on cross-competition with two V3 loop specific mAbs (HR10 and HGA9). This group comprised 8 mAbs, which were already mapped to the V3 loop using the Pepscan analysis approach, and 7 additional mAbs that were not analyzed by peptide scanning (i.e. HGF9, HGT4, HGP21, HGP51, HGW48, HGD129 and HGP27). Four mAbs (HX44, HGP105, HGW7, HGY38) reacted with wt and CD4bs YU2 mutants but failed to bind the CD4i mutant, and therefore were assigned to the CD4i cluster. Notably, one mAb (HGP68) was mapped by Pepscan analysis to a novel epitope in the V2 loop (TVYALFYRLDIVP) and neutralized 4 Tier-1 isolates (**Table S2**). Fifteen other mAbs were assigned tentatively or conclusively to the CD4bs based on their capacity to inhibit gp120-sCD4 binding. Most of these mAbs competed other CD4bs-specific mAbs, such as b12 and HJ16, while in some cases this assay could not be performed due to lack of epitope expression on the gp120 proteins used. Furthermore, this group of mAbs showed variable reactivities with the YU2 mutants. While most of them did not bind the D368R mutant similar to b12, others including HJ16, bound this mutant avidly. In addition, most of these new CD4bs-specific mAbs also showed decreased binding to the CD4i mutant, suggesting that they may span a broader, as yet undescribed region that includes elements of the CD4bs and CD4i sites. Finally, for 2 mAbs these analyses did not provide any relevant information.

Twenty-one mAbs bound to gp41 by targeting primarily the immunodominant C-C region, the HR-1 and the region recognized by 5F3 mAb [64] (**Table 2**). MAbs HGK129, HGN146 and HGN35 were assigned to the C-C loop since they competed with 3D6 [43], reacted with an HR2 construct and recognized synthetic peptides within this region. MAbs HGW17 and HGY25 were provisionally assigned to the C-C region since they competed with 3D6. These data are consistent with the immunodominance of the C-C region and with the notion that the antibody response against this region overlaps with the 3D6 epitope. Several mAbs competed with 5F3 and HK20, and bound both the HR-1 and 5HB constructs, suggesting that they may bind between the fusion peptide and the HR-1 region. HGB33 was provisionally assigned to the HR-1 region according to competition with HK20 and binding to the 5HB construct. Other mAbs bound specifically the 5HB construct but did not compete with any of the mAbs tested, thus indicating that the complete HR-1 coiled coil region exposed in 5HB harbors antibody epitopes available for B cell recognition in the gp41 pre-hairpin conformation [65], [66]. The concomitant binding to recombinant gp41 suggests that the latter may partially resemble the gp41 pre-fusion state. Of note, three gp41 binding mAbs (HGP40, HGP48 and HGY50) neither competed with any of the mAbs tested, nor bound to any constructs representing pre-hairpin conformations or native gp140 (**Figure S1**), indicating that they recognize as yet uncharacterized regions that are only available in the recombinant gp41 protein. Interestingly, mAb HGF24 was assigned by Pepscan analysis to an epitope in the C-terminal region of HR-2 (TNLIYTLIEESQN), proximal to the 2F5 epitope, and neutralized 4 Tier-2 isolates (**Table S2**). This mAb competed with HK20 for gp41 binding, in spite of their distinct cognate specificities (HR-2 and HR-1, respectively). This finding would be consistent with the proximity of HR-1 and HR2 in the six-helix bundle structure. Finally, although the gp41 protein used in the primary screening includes the 2F5 and 4E10 epitopes, no MPER specific mAbs were isolated, reinforcing the idea that this portion of gp41 is poorly immunogenic in humans [21], [22]

In conclusion, regardless of their limited breadth of neutralization, this extended panel of human mAbs may represent a useful tool for understanding the molecular basis of Env recognition in humans.

## Discussion

Using an improved EBV immortalization method combined with a broad screening strategy we isolated from memory B cells of HIV-1 infected donors 58 mAbs that cover an extensive antigenic surface of Env. Of these, 37 neutralized at least one of the isolates tested and 3 mAbs in particular bound to the CD4bs, V3 crown and HR-1, showing considerable neutralizing breadth against a panel of HIV-1 pseudoviruses of different clades and spanning Tier-1 and Tier-2 isolates.

Several studies have questioned the existence of individual broadly neutralizing antibody specificities as part of a normal immune response to HIV-1 infection. In this respect, b12 was isolated from a phage library, while 2F5, 4E10 and 2G12, although isolated from memory B cells, do not appear to have a counterpart in human sera [22]. In a recent study, Nussenzweig and coworkers used trimeric gp140 to isolate antigen-binding memory B cells from which 500 antibody sequences were retrieved by single-cell PCR [62]. Surprisingly, although the B cell donors had broadly neutralizing serum activity, none of the mAbs isolated had this property. This raised the possibility that broad serum neutralizing activity results from multiple clonal responses each of unique epitope specificity but restricted breadth of viral strain recognition. However, an alternative interpretation might be that since available recombinant trimeric antigens vary in structure, the ‘bait’ used for B cell isolation was not optimal for enriching those secreting neutralizing antibodies. Our results indicate that monoclonal antibodies with a limited spectrum of neutralizing activity can be easily isolated, while those with a broad pattern of cross-clade reactivity are rare, in line with the study of Walker et al [40] and consistent with inferences from serum neutralization specificity mapping analyses [21]. In this respect our study demonstrates the feasibility of the EBV immortalization method, which is limited only by the size of the blood samples obtained (20–50 ml of peripheral blood).

The characterization of the specificity and neutralizing activity of this new panel of human mAbs reveal some interesting features of the human antibody response to HIV-1 within a population of HIV-1-infected donors. First, most of the gp120-specific mAbs showed neutralizing activity (37 out of 58), indicating that they bind to sites available on the functional Env trimer. The selection of a high-percentage of trimer binding specificities may be a consequence our use of trimeric Env antigens during the screening of the EVB-B cell supernatants. The second aspect relates to gp41-specific antibodies, which, with a few remarkable exceptions, were not neutralizing. Interestingly the non-neutralizing antibodies bound gp41 in the context of the recombinant trimer, whereas the most effective neutralizing mAbs bound to gp41 fusion intermediates.

The three new neutralizing mAbs HJ16, HGN194 and HK20 show some interesting features. HJ16 showed a breadth of neutralizing activity comparable to, and generally complementary to b12, which aside from PG9 and PG16 [40] is currently the most potent of the relatively broad neutralizing antibodies available. HJ16 also showed selective neutralization of multiple Tier-2 isolates, making it particularly relevant as a template for vaccine design. HGN194 binds to an extremely conserved epitope in the V3 crown, and neutralizes all Tier-1, but only 11% of Tier-2 isolates tested. HGN194 showed broader activity than the well-characterized V3-specific mAb 447-52D against a panel of 92 pseudoviruses. The preference for Tier-1 isolates characteristic of V3-specific mAbs is consistent with the idea that the V3 loop is displayed to varying degrees in the context of the native trimeric Env protein of individual isolates [67], [68] and with the recent observation that sCD4 broadens neutralization of V3 mAbs [69]. The results obtained with HK20 are particularly intriguing, since this mAb has a broad pattern of neutralization observed with HOS but not TZMbl target cells. This mAb recognizes a highly conserved epitope in the HR-1 trimer and it is more effective as an Fab fragment as compared to intact IgG, a fact that is may be explained by the limited accessibility of the epitope, which is likely to be exposed only transiently on the cell surface during the viral entry process [70]. Intriguingly, a recent study indicates that in TZM-bl cells HIV-1 pseudoviruses penetrate the cytoplasm from within endosomes rather than from the cell surface [71], a finding that might help explain the lack of activity of HK20 in the TZMbl assay if binding of this mAb is affected by the endosomal environment. Further studies on primary cells will be required to address the relative importance of these two entry pathways and to establish whether HK20 may be capable of exerting a broad and potent neutralizing activity *in vivo*. Preliminary studies using M7 cells [52] challenged with replication-competent HIV-1 viruses carrying a Luc reporter showed that HK20 exhibits reasonably broad neutralization consistent with the results from the HOS cell assay, and similar to Fab HK20 in the TZM-bl cell assay. Whereas the TZM-bl assay is sensitive for certain antibodies such as CD4bs mAbs, some mAbs such as 2G12 to other epitopes are less sensitive to neutralization in this assay [72], perhaps owing to the unnaturally high level of CCR5 expression on the TZM-bl cells [60]. In this respect, low coreceptor expression levels have been associated with delayed fusion kinetics and hence enhanced virus sensitivity to HR-1 binding inhibitors, such as T-20 [73]. Therefore, despite the advantages of high throughput with the TZM-bl assay, alternative neutralization assays might have closer relevance to the *in vivo* situation.

In conclusion, we have shown that in HIV-1-infected individuals a high proportion of HIV-specific B cells produce neutralizing antibodies but only a few possess neutralizing activity with relatively broad neutralization coverage. In the short term, some of these reagents will be tested for their ability, singly or in combination, to prevent HIV-1 or SIV transmission in a macaque challenge model as done previously with other mAbs [5], [6], [7], [8], [9]. In the longer term, and combined with an atomic-level analysis of epitope specificity, some of these mAbs may facilitate the template-based design of immunogens for the development of a vaccine able to induce neutralizing antibodies against the wide range of HIV-1 strains present in the global pandemic.

## Supporting Information

Figure S1 Binding of gp120 and gp41-specific mAbs to a panel of 15 recombinant Env proteins from different clades.
(0.47 MB PDF)

Table S1 Clinical status of HIV-1 donors and HIV-1 neutralization breadth of plasma samples.
(0.42 MB PDF)

Table S2 MAb IC50 titers against HIV-1 in TZM-bl cells.
(0.10 MB PDF)

Table S3 Percentage of HIV-1 isolates neutralized in the TZM-bl based neutralization assay shown in Figure 2.
(0.27 MB PDF)

Table S4 HK20 Fab fragment shows increase in neutralization breadth and potency.
(0.23 MB PDF)

Table S5 MAb IC50 titers against HIV-1 M7-Luc cells.
(0.05 MB PDF)
