# Supplementary material for: Analysis of Memory B Cell Responses and Isolation of Novel Monoclonal Antibodies with Neutralizing Breadth from HIV-1-Infected Individuals
Source: PLoS One. 2010 Jan 20;5(1):e8805. doi: 10.1371/journal.pone.0008805 (PMC2808385; doi:10.1371/journal.pone.0008805)
Supplement: Figure S1 — Binding of gp120 and gp41-specific mAbs to a panel of 15 recombinant Env proteins from different clades. (0.47 MB PDF) [file pone.0008805.s001.pdf]

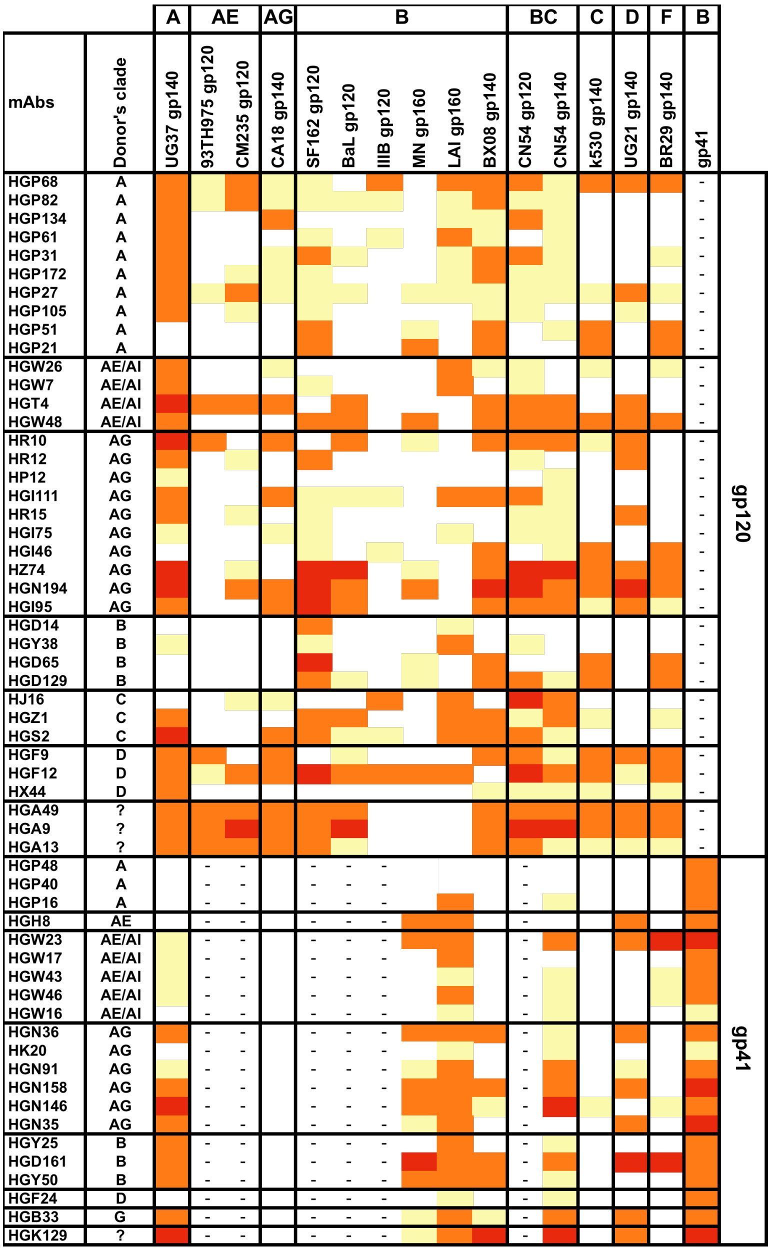

Figure S1. Binding of gp120 and gp41-specific mAbs to a panel of 15 recombinant Env proteins from different clades. Different dilutions of mAbs were tested in ELISA against a panel of recombinant Env proteins representing 8 different clades. The mAbs are grouped according to the donor's virus clade. Shown is the the half maximal binding concentration (K_{50}): red, 0.001-0.1 µg/ml, orange 0.1-5 µg/ml, yellow 5-500 µg/ml, white, no measurable binding.
