# Supplementary material for: Analysis of Memory B Cell Responses and Isolation of Novel Monoclonal Antibodies with Neutralizing Breadth from HIV-1-Infected Individuals
Source: PLoS One. 2010 Jan 20;5(1):e8805. doi: 10.1371/journal.pone.0008805 (PMC2808385; doi:10.1371/journal.pone.0008805)
Supplement: Table S1 — Clinical status of HIV-1 donors and HIV-1 neutralization breadth of plasma samples. (0.42 MB PDF) [file pone.0008805.s002.pdf]

Table S1. Clinical status of HIV-1 donors and HIV-1 neutralization breadth of plasma samples

									Serum reacts to					
Donor code	Cohort	Donor's clade	mAb code	Age	Years after infection / diagnosis	Viremia copies/ml	CD4 count	HAART	Clade A	Clade B	Clade C	CRFs	Clade D	Clade F
T650	London	D	HX	44	14.5	<40	518	2006- to date	na	na	0/2	2/3	na	1/1
VI 3208	Antwerp	C	HJ	45	12	<50	274	1997-to date	na	na	2/4	4/6	na	na
VI3265	Antwerp	CRF02_AG	HGN	41	10	125	765	Naïve	3/4	3/4	2/4	4/6	na	na
VI 3081	Antwerp	CRF02_AG	HK	31	1	150	623	Naïve	2/4	na	3/4	2/6	na	na
K500b	London	C	HGZ	40	4.5	1773	525	2005	na	0/2	2/3	0/4	0/1	0/1
D600	London	CRF01_AE	HGT	33	5.5	1821	707	2002	na	1/2	1/3	0/4	0/1	0/1
VI3229	Antwerp	CRF02/A1	HZ	43	20	6260	384	1997-2003	2/4	na	1/4	2/6	na	na
VI3207	Antwerp	A	HGP	40	11	26700	410	2006- to date	2/4	na	1/4	4/6	na	na
G400	London	B	HGY	40	13	27477	394	Naïve	na	0/2	2/3	1/4	0/1	0/1
VI3248	Antwerp	B	HGD	45	16	71000	373	Naïve	2/4	4/4	2/4	4/6	na	na
K300c	London	C	HGH	36	7	72901	362	Naïve	na	0/2	3/3	1/4	0/1	0/1
B420	London	CRF02_AG	HGI	37	4	86655	340	Naïve	na	1/2	2/3	0/4	0/1	0/1
A260	London	AE/AI	HGW	30	2.5	140434	362	Naïve	na	1/2	1/3	1/4	1/1	0/1
VI 3176	Antwerp	CRF02_AG	HP	32	7	300000	371	2006- to date	3/4	2/4	3/4	6/6	na	na
VI 3221	Antwerp	CRF02_AG	HY	31	6	330000	258	Naïve	3/4	2/4	4/4	6/6	na	na
VI 3186	Antwerp	CRF02_AG	HR	63	10	557000	259	2006- to date	1/4	na	1/4	4/6	na	na
m240	London	C	HGS	na	na	na	na	na	na	na	na	na	na	na
M320c	London	D	HGF	32	5	<50	311	Naïve	na	1/2	0/3	1/4	0/1	0/1
U225	London	G	HGB	na	na	na	na	na	na	0/2	0/3	1/4	0/1	1/1
R163	London	?	HGA	36	na	<1000	na	2002	na	1/2	1/3	0/4	0/1	0/1
S162	London	?	HGK	34	13	<40	416	Naïve	na	2/2	2/3	2/4	0/1	0/1

Shown is the donor code and cohort, the clade of infecting virus, the code assigned to the mAbs derived, clinical information and information regarding the state of the infection, therapy and breadth of reactivities of serum. na, not available.
