# Supplementary material for: Analysis of Memory B Cell Responses and Isolation of Novel Monoclonal Antibodies with Neutralizing Breadth from HIV-1-Infected Individuals
Source: PLoS One. 2010 Jan 20;5(1):e8805. doi: 10.1371/journal.pone.0008805 (PMC2808385; doi:10.1371/journal.pone.0008805)
Supplement: Table S2 — MAb IC50 titers against HIV-1 in TZM-bl cells. (0.10 MB PDF) [file pone.0008805.s003.pdf]

Table S2. MAb IC50 titers against HIV-1 in TZM-bl cells

Clade	Env clone	Tier	Year	Weeks after infection	Location	HGP105 (A)	HGP40 (A)	HGP16 (A)	HGP51 (A)	HGP21 (A)	HGP61 (A)	HGP172 (A)	HGP134 (A)	HGP31 (A)	HGP68 (A)	HGP82 (A)	HGW46 (AE)	HGT4 (AE)
A	MS208.A1	1	?	chronic	Montserrat	-	-	-	-	-	-	-	-	-	-	-	-	-
A	92RW020.2	1	1992	chronic	Rwanda	-	-	-	-	-	-	-	-	-	-	-	-	-
A	Q769.d22	2	?	Acute	Kenya	-	-	-	-	-	-	-	-	-	-	-	-	-
A	Q461.e2	2	?	Acute	Kenya	-	-	-	-	-	-	-	-	-	-	-	-	-
A	Q259.d2.17	2	?	Acute	Kenya	-	-	-	-	-	-	-	-	-	-	-	-	-
B	BaL.26	1	1985	chronic	USA	-	-	-	0.4	3.5	48.5	36.7	-	-	6.8	26.4	-	-
B	TRO.11	2	1995	4	Italy	-	-	-	-	-	-	-	-	-	-	-	-	-
B	SF162	1	?	chronic	San Francisco	-	-	-	-	16.3	4.7	18.2	46.1	14.8	12.4	2.6	-	-
B	MN-3	1	?	chronic	USA	-	-	-	-	0.1	0.2	0.9	6.4	0.5	-	0.1	-	-
B	WITO4160.33	2	2000	1	Alabama	-	-	-	-	-	-	-	-	-	-	-	-	-
B	PVO.4	2	1996	4	Italy	-	-	-	-	-	-	-	-	-	-	-	-	-
B	6535.3	2	1995	6	Washington D.C.	-	-	-	-	-	-	-	-	-	-	49.7	-	-
B	SC42261.8	2	1995	4	Trinidad	-	-	-	-	-	-	-	-	-	-	-	-	-
B	QH0692.42	2	1994	6	Trinidad	-	-	-	-	-	-	-	-	-	-	-	-	-
B	BX08.16	1	?	chronic	France	-	-	-	0.8	4.8	10.7	-	-	14.2	11.4	15.8	-	-
B	SS1196.1	1	1997	16	USA	-	-	-	0.4	5.7	16.0	35.7	-	-	-	26.8	-	-
B	RHPA4259.7	2	2000	<8	Tennessee	-	-	31.8	-	-	-	-	-	-	-	-	-	-
B	THRO4153.18	2	2000	1	Alabama	-	-	-	-	-	-	-	-	-	-	-	-	-
B	REJO4541.67	2	2001	2	Alabama	-	-	-	1.3	-	-	-	-	-	-	-	-	-
B	AC10.0.29	2	1998	4	Massachusetts	-	-	-	-	-	-	-	-	-	-	-	-	-
B	CAAN5342.A2	2	2004	<12	Alabama	-	-	-	-	-	-	-	-	nd	-	-	-	nd
B	TRJO4551.58	2	2001	1	Alabama	-	-	-	-	-	-	-	-	-	-	-	-	nd
C	93MW965.26	1	1993	?	Malawi	4.4	-	-	<0.02	-	1.2	0.1	0.4	0.1	0.1	0.1	-	<0.02
C	Du422.1	2	1998	8	Durban	-	-	-	-	-	-	-	-	-	-	-	-	-
C	CAP45.2.00.E8	2	2005	5	Durban	-	-	-	-	-	-	-	-	-	-	-	-	nd
C	Du156.12	2	1999	<4	Durban	-	-	-	-	-	-	-	-	-	-	-	-	-
C	Du172.17	2	1998	12	Durban	-	-	-	-	-	-	-	-	-	-	-	-	-
C	Du151.2	2	1998	6	Durban	-	-	-	-	-	-	-	-	-	-	-	-	-
C	ZM214M.PL15	2	2003	<13	Lusaka	-	-	-	-	-	-	-	-	-	-	-	-	-
C	ZM249M.PL1	2	2003	<1	Lusaka	-	-	-	-	-	-	-	-	-	-	-	-	nd
C	ZM197MPB7	2	2002	<15	Lusaka	-	-	-	-	-	-	-	-	-	-	-	-	-
C	CAP210.2.00.E8	2	2005	5	Durban	-	-	-	-	-	-	-	-	-	-	-	-	39.2
C	ZM53M.PB12	2	2000	<14	Lusaka	-	-	-	-	-	-	-	-	-	-	-	-	nd
C	ZM233M.PB6	2	2002	<15	Lusaka	-	-	-	-	-	-	-	-	-	-	-	-	-
C	ZM109F.PB4	2	2000	<14	Lusaka	-	-	-	-	-	-	42.3	43.1	-	-	-	-	-
C	96ZM651.2	2	1996	?	Zambia	-	-	-	-	-	-	-	-	-	-	-	-	-
C	ZM135M.PL10a	2	1998	<15	Lusaka	-	-	-	-	-	-	-	-	-	-	-	-	nd
AD	Q168.a2	2	1995	Acute	Kenya	-	-	-	-	-	-	-	-	-	-	-	-	nd
AG	263-8	2	?	chronic	W. Afr	-	-	-	-	-	46.1	-	-	-	-	-	-	nd
AG	211-9	2	?	Acute	Cameroon	-	-	-	-	-	-	-	-	-	-	-	-	nd
AG	T257-31	2	?	Acute	W. Afr.	-	-	-	-	-	-	-	-	-	-	-	-	-
AG	271-11	2	?	Acute	Cameroon	-	-	-	-	-	41.8	-	-	49.8	-	34.8	-	nd
BC	CH181.12	2	?	chronic	Beijing	-	-	-	-	-	-	-	-	-	-	-	-	-
BC	CH110.2	2	?	chronic	China	-	-	-	-	-	-	-	-	-	-	-	-	-
BC	CH064.20	2	?	chronic	Beijing	-	-	-	-	-	-	-	-	-	-	-	-	-
BC	CH111.18	2	?	?	China	-	-	-	-	-	-	-	-	-	-	-	-	-

Env clone	HGI46 (CRF02_AG)	HGI111 (CRF02_AG)	HGI75 (CRF02_AG)	HK20 (CRF02_AG)	HK20 Fab (CRF02_AG)	HR10 (CRF02_AG)	HR12 (CRF02_AG)	HZ74 (CRF02_AG)	HGI95 (CRF02_AG)	HGN36 (CRF02_AG)	HGN194 (CRF02_AG)	HP12 (CRF02_AG)
MS208.A1	-	-	-	38.2	6.9	24.4	-	40.1	-	-	8.6	-
92RW020.2	-	-	-	-	-	22.4	-	-	29.6	-	5.6	-
Q769.d22	-	-	-	31.5	-	-	-	-	-	-	-	-
Q461.e2	-	-	-	-	8.8	-	-	-	-	-	-	-
Q259.d2.17	-	-	-	-	-	-	-	46.4	-	-	46.5	-
BaL.26	8.7	49.3	-	-	-	14.7	-	0.6	12.1	-	0.1	-
TRO.11	-	-	-	-	-	-	-	-	-	-	-	-
SF162	49.6	13.4	-	-	-	-	-	0.1	0.1	-	<0.02	-
MN-3	1.9	1.1	9.2	-	0.4	<0.02	44.9	0.0	0.0	-	<0.02	-
WITO4160.33	-	-	-	-	-	-	-	-	-	-	-	-
PVO.4	-	-	-	-	-	-	-	-	-	-	-	-
6535.3	8.9	-	-	-	-	-	-	3.4	-	-	-	-
SC42261.8	-	-	-	-	-	-	-	-	-	-	-	-
QH0692.42	-	-	-	-	-	-	-	-	-	-	-	-
BX08.16	26.1	13.5	-	-	-	3.2	3.5	0.3	6.3	-	0.1	-
SS1196.1	-	-	-	-	-	27.3	-	2.9	3.7	-	0.6	-
RHPA4259.7	-	-	-	-	-	-	-	-	-	-	35.4	-
THRO4153.18	-	-	-	-	-	-	-	20.4	-	-	-	-
REJO4541.67	-	-	-	-	-	-	-	-	-	-	14.2	-
AC10.0.29	-	-	-	-	-	-	-	-	-	-	-	-
CAAN5342.A2	-	-	-	-	nd	-	-	-	-	-	-	-
TRJO4551.58	-	-	-	-	nd	-	-	-	-	-	-	-
93MW965.26	-	-	0.6	17.3	1.6	<0.02	-	<0.02	<0.02	-	<0.02	-
Du422.1	-	-	-	-	6.5	-	-	-	-	-	-	-
CAP45.2.00.E8	-	-	-	-	nd	-	-	-	-	-	-	-
Du156.12	-	-	-	-	7.3	-	-	-	-	-	-	-
Du172.17	-	-	-	-	5.2	-	-	-	-	-	-	-
Du151.2	-	-	-	-	-	-	-	-	-	-	-	-
ZM214M.PL15	-	-	-	-	nd	-	-	-	-	-	-	-
ZM249M.PL1	-	-	-	-	nd	-	-	-	-	-	-	-
ZM197MPB7	-	-	-	-	-	-	-	-	-	-	-	-
CAP210.2.00.E8	-	-	-	-	5.8	-	-	-	-	-	-	-
ZM53M.PB12	-	-	-	-	nd	-	-	-	-	-	-	-
ZM233M.PB6	-	-	-	-	nd	-	-	48.7	-	-	10.5	-
ZM109F.PB4	-	-	-	-	-	7.2	-	-	-	-	2.2	-
96ZM651.2	-	-	-	-	-	-	-	-	-	-	-	-
ZM135M.PL10a	-	-	-	-	nd	-	-	-	-	-	-	-
Q168.a2	-	-	-	-	nd	-	-	-	-	-	-	-
263-8	-	-	-	-	nd	-	-	-	-	-	-	-
211-9	-	-	-	-	nd	-	-	-	-	-	-	-
T257-31	-	-	-	-	-	-	-	-	-	-	-	-
271-11	-	-	-	-	nd	0.1	-	0.3	0.5	-	0.1	-
CH181.12	-	-	-	-	-	-	-	-	-	-	49.6	-
CH110.2	-	-	-	-	5.8	-	-	-	-	-	-	-
CH064.20	-	-	-	-	nd	-	-	-	-	-	-	-
CH111.18	-	-	-	-	5.7	-	-	-	-	-	-	-

Env clone	HGD14 (B)	HGD161 (B)	HGD65 (B)	HGD129 (B)	HGY50 (B)	HGH8 (C)	HGS2 (C)	HGZ1 (C)	HJ16 (C)	HGF12 (D)	HX44 (D)	HGF24 (D)	HGF9 (D)	HGB33 (F)	HGA49 (nd)	HGA9 (nd)
MS208.A1	-	-	-	-	-	-	-	-	-	-	-	-	30.5	-	50.0	36.2
92RW020.2	-	-	-	-	-	-	-	-	1.9	-	-	-	-	-	-	46.4
Q769.d22	-	-	-	-	-	-	-	-	-	-	-	-	-	-	-	-
Q461.e2	-	-	-	-	-	-	-	-	0.2	-	-	-	-	-	-	-
Q259.d2.17	-	-	-	-	-	-	-	-	-	-	-	-	-	-	-	-
BaL.26	15.0	-	0.4	0.3	-	-	-	31.3	-	-	-	-	-	-	12.7	7.0
TRO.11	-	-	-	-	-	-	-	-	0.1	-	-	-	-	-	-	-
SF162	4.2	-	0.1	0.2	-	-	18.2	2.5	-	-	-	-	-	-	1.2	11.4
MN-3	1.9	-	<0.02	<0.02	-	-	2.8	0.2	-	-	17.8	-	-	-	0.3	0.5
WITO4160.33	-	-	-	-	-	-	-	-	-	-	-	-	-	-	-	-
PVO.4	-	-	-	-	-	-	-	-	14.5	-	-	-	-	-	-	-
6535.3	10.5	-	2.9	12.2	-	-	-	12.4	-	-	-	-	-	-	-	-
SC42261.8	-	-	-	-	-	-	-	-	31.1	-	-	-	-	-	-	-
QH0692.42	-	-	-	-	-	-	-	-	16.4	-	-	-	-	-	-	-
BX08.16	-	-	0.5	0.2	-	-	22.1	5.7	-	-	-	-	44.0	-	1.9	39.0
SS1196.1	-	-	3.4	1.6	-	-	-	50.0	-	-	-	-	-	-	16.1	-
RHPA4259.7	-	-	-	-	-	-	-	-	<0.02	-	-	-	-	-	-	-
THRO4153.18	-	-	-	-	-	-	-	-	-	-	-	-	-	-	-	-
REJO4541.67	23.2	-	-	12.7	-	-	-	-	-	-	-	-	-	-	-	-
AC10.0.29	-	-	-	-	-	-	-	-	-	-	-	-	-	-	-	-
CAAN5342.A2	-	-	-	-	-	-	-	-	11.2	-	-	-	-	-	-	-
TRJO4551.58	-	-	-	-	-	-	-	-	-	-	-	-	-	-	-	-
93MW965.26	-	-	-	-	-	-	3.3	<0.02	-	-	0.3	-	0.04	-	0.1	<0.02
Du422.1	-	-	-	-	-	-	-	-	-	-	-	-	-	-	-	-
CAP45.2.00.E8	-	-	-	-	-	-	-	-	-	-	-	-	-	-	-	43.3
Du156.12	-	-	-	-	-	-	-	-	-	-	-	-	-	-	-	-
Du172.17	-	-	-	-	-	-	-	-	23.7	-	-	-	-	-	-	-
Du151.2	-	-	-	-	-	-	-	-	-	-	-	-	-	-	-	-
ZM214M.PL15	-	-	-	-	-	-	-	-	0.1	-	-	-	-	-	-	-
ZM249M.PL1	-	-	-	-	-	-	-	-	-	-	-	-	-	-	-	-
ZM197MPB7	-	-	-	-	-	-	-	-	-	-	-	-	-	-	-	-
CAP210.2.00.E8	-	-	-	-	-	-	-	35.5	-	-	-	-	36.1	-	-	-
ZM53M.PB12	-	-	-	-	-	-	-	-	-	-	-	37.6	-	-	-	-
ZM233M.PB6	-	-	-	-	-	-	-	-	-	-	-	32.7	36.4	-	-	42.7
ZM109F.PB4	-	-	-	-	-	-	-	48.2	2.9	-	-	-	-	-	-	14.6
96ZM651.2	-	-	-	-	-	-	-	-	0.2	-	-	-	-	-	-	-
ZM135M.PL10a	-	-	-	-	-	-	-	-	-	-	-	45.2	-	-	-	-
Q168.a2	-	-	-	-	-	-	-	-	0.04	-	-	-	-	-	-	-
263-8	-	-	-	-	-	-	-	-	-	-	-	31.0	-	-	-	-
211-9	-	-	-	-	-	-	-	-	3.9	-	-	-	-	-	-	-
T257-31	-	-	-	-	-	-	-	-	17.0	-	-	-	-	-	-	-
271-11	-	-	0.8	-	-	-	-	47.3	0.1	-	-	-	0.2	-	0.3	0.5
CH181.12	-	-	-	-	-	-	-	-	43.9	-	-	-	-	-	-	-
CH110.2	-	-	-	-	-	-	-	-	-	-	-	-	-	-	-	-
CH064.20	-	-	-	-	-	-	-	-	-	-	-	-	-	-	-	-
CH111.18	-	-	-	-	-	-	-	-	4.9	-	-	-	-	-	-	-

MAbs were purified and tested at different concentrations starting from 50 μg/ml for their capacity to neutralize 46 HIV-1 pseudoparticles representing 6 different clades. Shown are IC50 values (μg/ml). Black cases, 0.01-1 μg/ml; dark grey, 1.1-10 μg/ml; light grey, 10.1-50 μg/ml; MAbs neutralizing <50% at 50 μg/ml were scored as negative (-).
