# Supplementary material for: Analysis of Memory B Cell Responses and Isolation of Novel Monoclonal Antibodies with Neutralizing Breadth from HIV-1-Infected Individuals
Source: PLoS One. 2010 Jan 20;5(1):e8805. doi: 10.1371/journal.pone.0008805 (PMC2808385; doi:10.1371/journal.pone.0008805)
Supplement: Table S3 — Percentage of HIV-1 isolates neutralized in the TZM-bl based neutralization assay shown in Figure 2. (0.27 MB PDF) [file pone.0008805.s004.pdf]

Table S3. Percentage of HIV-1 isolates neutralized in the TZM-bl based neutralization assay shown in Figure 2

		HK20	HGN194	HJ16	b12	2G12	2F5	4E10	447-52D
	All (92)	3%	21%	36%	47%	28%	39%	98%	11%
	Tier 1 (10)	20%	100%	10%	80%	67%	67%	100%	88%
	Tier 2 (82)	1%	11%	39%	43%	23%	36%	99%	4%
Tier-1+Tier-2	Clade A (8)	25%	38%	25%	25%	13%	88%	100%	0%
	Clade AD (1)	0%	0%	100%	0%	0%	100%	100%	0%
	Clade AG (16)	0%	0%	50%	25%	38%	25%	100%	0%
	Clade B (29)	0%	24%	34%	69%	55%	79%	97%	28%
	Clade BC (11)	0%	0%	45%	36%	18%	0%	100%	0%
	Clade C (26)	4%	23%	27%	54%	0%	8%	100%	4%
	Clade G (1)	0%	0%	0%	0%	0%	0%	100%	0%
Tier-2	Clade A (5)	14%	20%	20%	0%	0%	100%	100%	0%
	Clade AD (1)	0%	0%	100%	0%	0%	100%	100%	0%
	Clade AG (16)	0%	0%	50%	25%	38%	25%	100%	0%
	Clade B (24)	0%	8%	42%	63%	50%	75%	97%	13%
	Clade BC (11)	0%	0%	45%	36%	18%	0%	100%	0%
	Clade C (24)	0%	17%	29%	54%	0%	9%	100%	0%
	Clade G (1)	0%	0%	0%	0%	0%	0%	100%	0%

Shown is the percentage of HIV-1 isolates neutralized by each mAb as indicated in Figure 2. In parenthesis are shown the number of isolates in each analyzed group.
