# Supplementary material for: Analysis of Memory B Cell Responses and Isolation of Novel Monoclonal Antibodies with Neutralizing Breadth from HIV-1-Infected Individuals
Source: PLoS One. 2010 Jan 20;5(1):e8805. doi: 10.1371/journal.pone.0008805 (PMC2808385; doi:10.1371/journal.pone.0008805)
Supplement: Table S4 — HK20 Fab fragment shows increase in neutralization breadth and potency. (0.23 MB PDF) [file pone.0008805.s005.pdf]

Table S4. HK20 Fab fragment shows increase in neutralization breadth and potency

		IC50 (µg/ml)	
**HIV-1 isolate**	**Clade**	**IgG**	**Fab**
**Q461.e2**	A	7.34	0.49
**Q769.d22**	A	9.22	0.11
**Q168.a2**	AD	40.85	0.12
**T257-31**	AG	35.04	0.16
**263-8**	AG	68.70	1.19
**SF162**	B	9.64	0.27
**SC42261.8**	B	10.03	0.15
**CAAN5342.A2**	B	9.63	0.09
**JRFL**	B	>100	>10
**BaL**	B	84.77	0.28
**THRO4153.67**	B	>100	0.48
**H022.7**	B	>100	5.70
**CH181.12**	BC	15.50	0.43
**CH064.20**	BC	11.10	0.06
**ZM214M.PL15**	C	5.84	0.01
**ZM53M.PB12**	C	15.44	0.60
**ZM109F.PB4**	C	14.14	0.15
**CAP210.2.00.E8**	C	11.89	0.16
**Du151..2**	C	16.62	0.61
**ZM249.PL1**	C	6.68	0.25
**93MW965.26**	C	8.50	0.08
**96ZM651.2**	C	1.46	0.04
**VSV-G**		>100	>10

HK20 IgG and Fab fragments were tested against a panel of 28 HIV-1 primary isolates in a HOS-based neutralization assay. Shown is the IC50 value in µg/ml.
