# Supplementary material for: Analysis of Memory B Cell Responses and Isolation of Novel Monoclonal Antibodies with Neutralizing Breadth from HIV-1-Infected Individuals
Source: PLoS One. 2010 Jan 20;5(1):e8805. doi: 10.1371/journal.pone.0008805 (PMC2808385; doi:10.1371/journal.pone.0008805)
Supplement: Table S5 — MAb IC50 titers against HIV-1 M7-Luc cells. (0.05 MB PDF) [file pone.0008805.s006.pdf]

Table S5. MAb IC50 titers against HIV-1 M7-Luc cells

	ID50 (mg/ml) in M7-Luc cells				
mAb	Bal.LucR.T2A.ecto	SF162.LucR.T2A.ecto	CH40.LucR.T2A.ecto	CH58.LucR.T2A.ecto	WITO.LucR.T2A.ecto
HK20	22.7	3.2	3.9	24.3	-*
HGN194	7.1	<0.05	-	2.1	-*
HJ16	-	-	-	-*	-*
Ctr mAb	-	-	-	nd	nd

MAbs were purified and tested at different concentrations starting from 100 µg/ml for their capacity to neutralize 5 HIV-1 replication competent infectious viruses (clade B) carrying luciferase gene as reporter gene and using M7-Luc cells as target cells. Shown are IC50 values (µg/ml). Red cases, 0.01-1 µg/ml; orange, 1.1-10 µg/ml; yellow, 10.1-100 µg/ml; MAbs neutralizing <50% at 100 µg/ml were scored as negative (-). -*, mAbs tested at 50 µg/ml.nd, not tested.
